# Potential Sustainability and Stress Resilience of Forest Trees Induced by Nanotechnology Applications

**DOI:** 10.1002/pei3.70105

**Published:** 2025-12-15

**Authors:** Abubakr M. J. Siam, Rund Abu‐Zurayk, Rehab M. Abdelkheir, Nasreldeen Siam, Rida Shibli, Jamal Sawwan

**Affiliations:** ^1^ Department of Forestry & Range Science, Faculty of Environmental Sciences & Natural Resources University of Al Fashir El Fasher Sudan; ^2^ Department of Horticulture & Crop Science, Faculty of Agriculture University of Jordan Amman Jordan; ^3^ Hamdi Mango Center for Scientific Research The University of Jordan Amman Jordan; ^4^ Department of Forestry, College of Natural Resources & Environmental Studies University of Bahri Khartoum Sudan; ^5^ Department of Industrial Chemistry, College of Applied & Industrial Science University of Bahri Khartoum Sudan

**Keywords:** environmental stressors, nanoparticles, nanotechnology, reforestation, sustainable forestry, tree growth

## Abstract

Forest ecosystems play a crucial role in mitigating climate change, conserving biodiversity, supporting bioenergy production, and providing green jobs that sustain the livelihoods of billions worldwide. However, in recent decades, forests have become increasingly vulnerable to a range of abiotic and biotic stresses that impede forest yield and development, thereby threatening environmental stability, food security, and global human well‐being. Key challenges include climate change, water scarcity, soil‐related constraints, overcutting, and pathogenic infestations, all of which hinder successful growth and productivity. Emerging nanotechnology, particularly the application of nanoparticles (NPs) ranging from 1 to 100 nm, offers innovative solutions in forestry. This review analyzes published research over the past 25 years on the applications of NPs in forest production, with a particular focus on enhancing reforestation efforts and stress resilience. Out of the 64 researches reviewed, the key areas of investigation include improvements in seed germination (14%), plant growth (36%), and physiological tolerance to drought (18.6%), salinity and toxicity (9.7%), pests and diseases (8.6%), and wildfire stressors (13%). Approximately 97% of NP applications have shown beneficial effects on critical growth and physiological parameters, although a small number of studies report adverse outcomes. Future applications in forestry should emphasize the optimization of commonly used NPs, including silver (AgNPs), zinc oxide (ZnO NPs), silicon dioxide (SiO_2_ NPs), and iron‐based NPs. Notably, the current literature remains limited in its coverage of global tree species. This review advocates for a synergistic, interdisciplinary approach to advance the sustainable integration of nanotechnology into forestry practices and to broaden its application across a wider diversity of tree species. Collaborative research efforts will be essential to further develop this promising field.

## 1 Introduction

Forests cover about one‐third of the Earth's land area comprising diverse forest types shaped by climatic zones and tree species (WWF 2018). They provide more than 86 million green jobs and support the livelihoods of over two billion people worldwide (UNECE 2018; Jin 2025). Forest ecosystems play a vital role in mitigating climate change, conserving biodiversity, supporting bioenergy stock, and enhancing societal well‐being, thereby contributing to the achievement of the Sustainable Development Goals (Niu et al. 2023; Raman et al. 2024; Psistaki et al. 2024). However, in recent decades, various abiotic and biotic factors have posed major challenges to the sustainable growth and productivity of forest ecosystems. These threats include climate change, water stress, soil degradation, overcutting, and disease, all of which jeopardize environmental stability, food security, and human welfare on a global scale (Contreras‐Hermosilla 2000; Njana et al. 2021; Madalcho et al. 2020; Sharma et al. 2017; Brack 2019; Dockry et al. 2020; Bussotti and Pollastrini 2021). Forests face increasing pressures from the combined effects of climate change and the growing global demand for forest products (IUFRO 2024). Between 1990 and 2020, the world lost approximately 178 million hectares of forest, an area roughly equivalent to the size of Libya (FAO 2020). In response, forestry and agroecology researchers, managers, and policymakers are urged to develop and implement sustainable solutions for forest conservation and management (Abdelkheir et al. 2011; Kubik 2020; Abas et al. 2022; Wagay et al. 2023; Budiman and Ou 2025; Ramli et al. 2025). These efforts align with the Global Forest Goals (GFGs) of the United Nations Strategic Plan for Forests (2017–2030), which emphasizes the context of climate change and sustainable forest management (Brack 2019).

**FIGURE 1:**
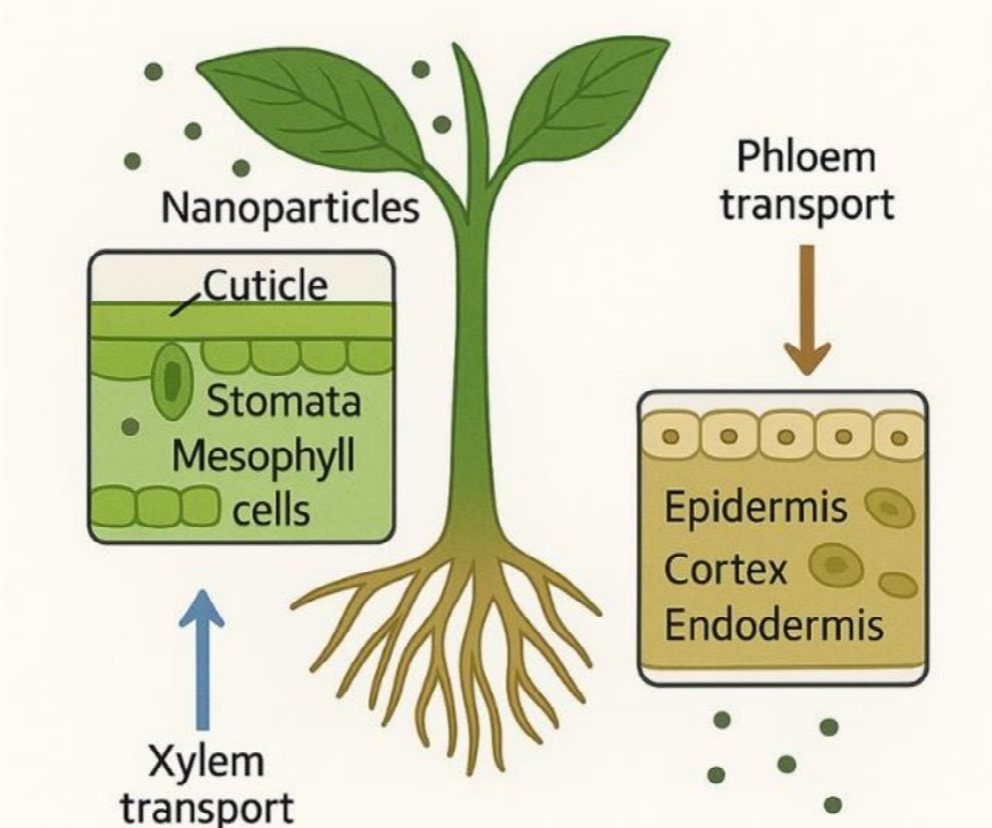
Foliar and root uptake and translocation of nanoparticles in plant.

One promising avenue for enhancing forest resilience to natural and anthropogenic pressures is the application of cutting‐edge technologies such as nanotechnology. This interdisciplinary field involves the development and use of nanoscale materials (1–100 nm) termed nanoparticles (NPs) and has demonstrated potential in enhancing plant growth, seed germination, and environmental adaptability (Chausali et al. 2023; Disfani et al. 2017; Ralia et al. 2018; Shibli et al. 2022; Shah et al. 2024; Abdalkreem et al. 2024; Mohusaien et al. 2024). In agriculture, nanotechnology has already introduced innovations such as nano‐fertilizers, nano‐pesticides, and precision farming tools, contributing to improved productivity and ecological balance (Du et al. 2019; Usman et al. 2020; Chausali et al. 2023; Quintarelli et al. 2024). Growth characteristics, physiological parameters, and resistance to abiotic and biotic stresses could be promoted by nanotechnological applications (Ashkavand, Tabari, et al. 2018; Mehmood et al. 2021). As illustrated in Figure 1, uptake and translocation of nanoparticles through root and foliar pathways noticeably enhance plant growth and physiological parameters (Ali et al. 2021; Nile et al. 2022).

Nanoparticles show promise in advancing sustainable agroecological practices and enhancing productivity (Cheng et al. 2016; Al‐Qudah et al. 2022; Irewale et al. 2024). Despite these prospects, research on the application of nanotechnology in forest trees and woody plants remains limited (Wagay et al. 2023). Thus, there is a pressing need to review and analyze existing literature to explore the potential impacts and identify research gaps of nanotechnology in forestry. It is important to acknowledge that nanotechnology may also pose risks to the environment and living organisms. Engineered nanoparticles can interact with agroforestry systems at molecular and physiological levels, potentially causing nanotoxicity (Ghormade et al. 2011; Chichiricco and Poma 2015; Armstead and Li 2016) and impacting human health (Bhardwaj et al. 2022; Verma et al. 2022; Fu et al. 2014; Bratovcic et al. 2021). Therefore, future research should not only focus on maximizing the benefits of nanomaterials but also on understanding and mitigating their potential health and environmental risks, thereby providing a comprehensive foundation for their safe and effective use in agriculture and forestry (Singh, Sillu, et al. 2021; Irewale et al. 2024; Siam et al. 2025).

## 2 Nanotechnology in Forestry

**FIGURE 2:**
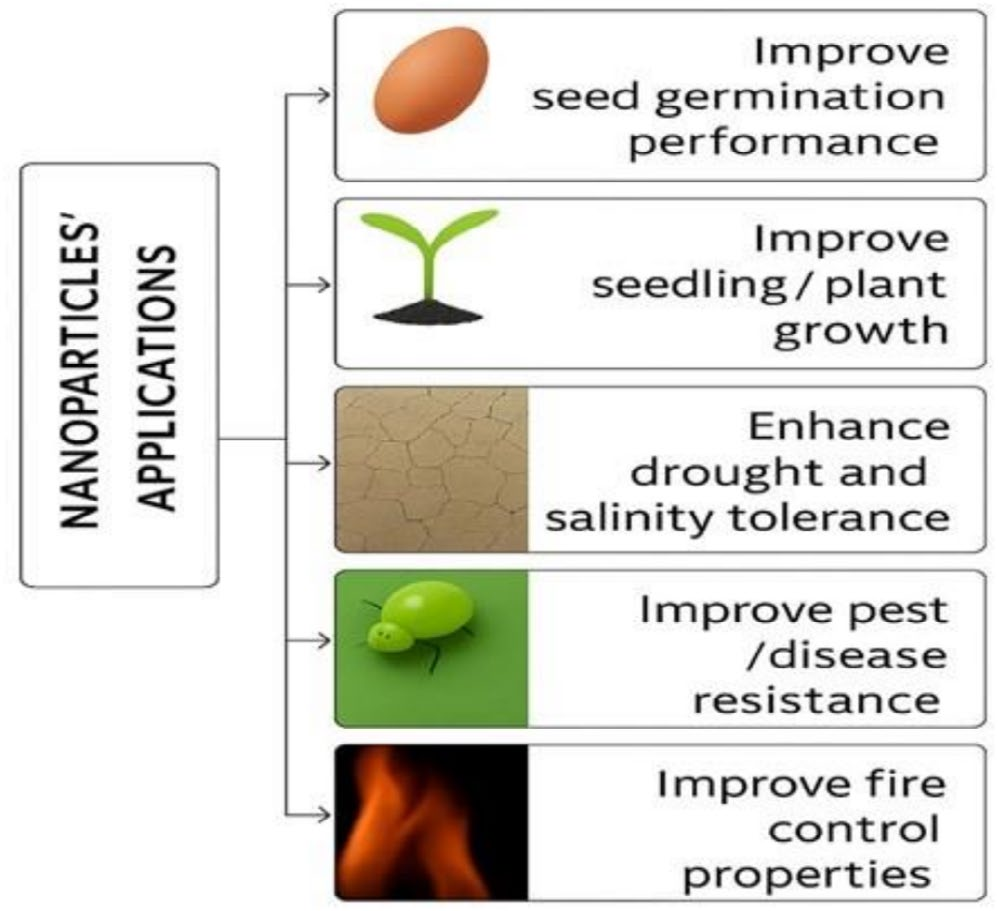
Positive effects of nanoparticles' applications in forest tree performance.

As forests increasingly face tremendous strains due to climate change and rising demands for forest products, nanotechnology has emerged as a valuable tool across various forestry‐related fields, including seed germination, tree growth, forest management, harvesting, and wood quality enhancement (McCrank 2009; Garza‐Alonso et al. 2021; Bi et al. 2021). Nanotech offers innovative solutions to numerous challenges within the forestry sector, such as improving and protecting wood quality, aiding in forest fire suppression, promoting successful seed germination, and enhancing the delivery of herbal medicines (Qi et al. 2013; Singh et al. 2015; Surendra et al. 2023; Babali et al. 2024). As illustrated in Figure 2, nanotechnology serves as a promising input for advancing silvicultural practices and forest protection activities. The forest ecosystem serves as an essential application hub and fundamental biogenic source for green synthesis of nanomaterials (Ali et al. 2020; Siam et al. 2025). Despite this, the body of literature focused on nanotechnology applications in forestry remains limited (Singh, Arya, et al. 2021; Wagay et al. 2023). This review aims to survey and analyze research conducted over the last 25 years on the use of nanotechnology in forestry. Specifically, it seeks to highlight the outcomes of nanotech applications in silvicultural practices such as seed germination and tree growth, and in mitigating environmental and biological stresses, including drought, salinity, toxicity, pests, diseases, and wildfires.

### 2.1 Silviculture

Silviculture is the practice of controlling the establishment, growth, composition, and quality of forests to meet the needs and values of societies on a sustainable basis (Waring and Bucholz 2023). Establishment of a forest in an area that was previously not occupied by forest trees is known as afforestation, while reforestation means replanting of trees in an area that was forest and has been degraded or deforested. Many countries have ambitious programs of afforestation and reforestation, but they are still facing poor germination and early growth challenges (Shi et al. 2010; Grossnickle and Ivetic 2017; Jalonen et al. 2017; Zhou et al. 2022). Successful germination and good early growth of seedlings are prerequisites for plantation success (Elmagboul et al. 2014; Abdullah and Siam 2017; Lamhamedi et al. 2023). Recently, the applications of nanoparticles improved tree planting opportunities and success through enhancement of seedling survival and growth of Scot pines (Ayan et al. 2023), germination and growth capacity of acacia tree species (Abdalkreem et al. 2024, 2025), and increased root mycorrhization in *Quercus robur* (Olchowik et al. 2017).

#### 2.1.1 Seed Germination

Understanding of tree seed germination characteristics represents a cornerstone for the successful establishment of forest stands (Nakar and Jadeja 2014; Dias Laumann et al. 2023). One of the central processes in ensuring the success of reforestation activities is the germination capacities of the respective seeds (Adelani 2015; Francheteau 2024). Optimal germination is a critical part for the successful establishment of trees and crops (Adelani et al. 2021; Ghaleb et al. 2022). Therefore, pre‐treatment in many cases is necessary to activate germination of required seeds (Chauhan et al. 2009; Om et al. 2020), especially in forest tree species where the seed germination is low due to hard seed coats and dormancy (Elmagboul et al. 2014; Ahmed et al. 2017; Abdullah and Siam 2017). As shown in Table 1, numerous studies in recent years have explored the use of nanotechnology as a possible pre‐sowing treatment or nano‐fertilizers (Siddique and Bose 2007; Dasgupta et al. 2017; Krishnaraj et al. 2012). Priming of seeds has brought significance and interest among scientific communities, thereby inducing changes in seed metabolism leading to rapid and higher germination and seedling growth, which could support the establishment of plants in their environment faster and healthier (Kandhol et al. 2022). Various metal nanoparticles, such as silver, silicon, iron, and copper nanoparticles, act as potent catalysts for breaking seed dormancy and enhancing germination traits in various tree and bamboo species (Savithramma et al. 2012; Azeez et al. 2019; Polischuk et al. 2019; Zhou et al. 2022). Moreover, metal oxide and carbon nanoparticles revealed promotion of seed germination in various tree and other plant species (Ali et al. 2020; Garza‐Alonso et al. 2021; Emamverdian et al. 2021; Zhou et al. 2022; Sarkhosh et al. 2022).

**TABLE 1:** Nanotechnology products applications in seed germination of forest and woody plant species.

Nanoparticles (NPs)	Concentration of NPs	Effects on tree	Tree species	References
Carbon nanoparticles functionalized with carboxylic acid (MWCNT–COOH)	20 and 40 μg	Resolved dormancy and improved germination rate of seeds	*Alnus viridis* & *Shepherdia canadensis*	Ali et al. 2020
Zinc Oxide nanoparticles (ZnO NPs)	0.5, 2.5, 5, 7.5 and 10 mg L^−^1	Increased seed germination by 25%–30%	*Moringa oleifera*	Garza‐Alonso et al. 2021
Silver Nanoparticles (AgNPs)	25, 50, and 75 mg L^−1^	Broke dormancy, increased germination percentage and speed of seeds	*Acacia senegal & Acacia mellifera*	Abdalkreem et al. 2024
AgNPs	0.2 mg	Improved germination percentages of seeds	*Moringa oleifera*	Azeez et al. 2019
AgNPs	10–30 μg/mL	Increased germination percentage and speed of seeds	*Boswellia ovalifoliolata*	Savithramma et al. 2012
AgNPs	0, 10, 20, 40, 80 and 100 mg/soil kg & 0, 10, and 20 mg/L of water	No effect detected for germination in low concentrations. 80 and 100 mg/kg & 10 mg/L decreased percentage and speed of germination of seeds	*Pinus sylvestris*	Bayramzadeh et al. 2019
Iron and copper nanoparticles (FeNPs & CuNPs)	2.10^−4^%—2.10^−2^%	Increased germination to 10% above control of seeds	*Pinus sylvestris*	Polischuk et al. 2019
Liquid Phase Nano Titanium Dioxide (TiO_2_)	50, 100, 200 and 500 mg/L	Promoted seed germination rate, potential and index. excessive concentration brought some side effects on germination	Camphor tree ( *Cinnamomum camphora* )	Zhou et al. 2022
AgNPs	200, 400, 600, 800, and 1000 mg/L	Caused decrease in the rate and percentage of seed germination	*Fagus orientalis*	Ozel et al. 2024

On the other hand, negative effects of nanoparticles on germination of *Fagus orientalis* tree seeds have been reported (Ozel et al. 2024). High concentrations of nanoparticles often bring side effects in seed germination as shown in *Pinus sylvestris* (Bayramzadeh et al. 2019) and *Cinnamomum camphora* trees (Zhou et al. 2022). More research in the field of nanotechnology applications in seed germination is needed to assess the potential beneficial and harmful effects in various forest tree species.

#### 2.1.2 Seedling and Tree Growth

Nano‐fertilizers are recognized as crucial elements for improving nutrient use efficiency, soil properties, plant growth, yield, and biomass production thus reducing cultivation costs (Bao‐shan et al. 2004; Ashkavand et al. 2015; El‐Kady et al. 2017; Azeez et al. 2019; Sarkhosh et al. 2022; Wagay et al. 2023). Using nano‐fertilizers instead of traditional chemical‐based fertilizers could increase agricultural outputs by up to 30% (Jyothi and Hebsur 2017; Kalia and Sharma 2019). Characteristics such as shoot height, leaf number, root length, growth index, and dry matter production are largely improved by applications of nanoparticles as shown in Table 2. Nanoparticles of silver, silicon, copper, and iron have been shown to promote survival rates, mycorrhization, and growth characteristics in various tree species, including *Acacia*, *Crataegus*, *Moringa*, *Boswellia*, *Quercus*, *Pinus*, *Populus*, and *Fagus* (Savithramma et al. 2012; Ashkavand et al. 2015; Olchowik et al. 2017; Aleksandrowicz‐Trzcinska et al. 2018, 2019; Polischuk et al. 2019; Azeez et al. 2019; Vasyukova et al. 2021; Abdalkreem et al. 2024). Application of nanostructured silicon dioxide and carbon nanoparticles functionalized with carboxylic acid (MWCNT–COOH) improved growth vigor and chlorophyll contents in *Larix olgensis, Alnus viridis*, and *Shepherdia canadensis* tree seedlings (Bao‐shan et al. 2004; Ali et al. 2020). Furthermore, it has been reported that the dry biomass of *Betula* tree leaf, stem, and root increased by 30%, 42%, and 49%, respectively, when used Multi‐Walled Carbon Nanotubes (MWCNTs) at a concentration of 10 mg/L (Zhuzhukin et al. 2023). Scots pine ( *Pinus sylvestris* ), *Moringa peregrina*, *Moringa oleifera* , *Photinia fraseri*, *Cotinus coggygria* , and camphor ( *Cinnamomum camphora* ) trees treated by zinc, iron, titanium, copper, and silicon oxides nanoparticles exhibited higher growth, fresh weight, photosynthetic pigments, and bioactive compounds (Soliman et al. 2015; Garza‐Alonso et al. 2021; Zhou et al. 2022; Ayan et al. 2023; Babali et al. 2024).

**TABLE 2:** Nanotechnology products applications in growth of forest and woody plant species.

Nanoparticles (NPs)	Concentration of NPs	Effects on tree	Tree species	References
Silver nanoparticles (AgNPs)	25, 50, and 75 mg L^−1^	Enhanced survival rate and height of shoot and root of seedlings	*Acacia senegal & Acacia mellifera*	Abdalkreem et al. 2024
Silicon oxide nanoparticles (SO_2_ NPs)	10, 50, and 100 mg L^−1^	Increase biomass of seedlings	hawthorns tree ( *Crataegus monogyna* )	Ashkavand et al. 2015
TMS (nanostructured silicon dioxide)	2000 μL L^−1^ 1000 μL L^−1^ 500 μL L^−1^ 250 μL L^−1^ 125 μL L^−1^ 62 μL L^−1^	Improvement of seedling height and chlorophyll content, root diameter, root length, number of lateral roots	*Larix olgensis*	Bao‐shan et al. 2004
AgNPs	0.2 mg	Improved shoot and root growth and leaf number of seedlings	*Moringa oleifera*	Azeez et al. 2019
Multi‐walled carbon nanotubes (MWCNTs)	1, 10, 50, and 100 mg/L	Increased length and diameter of shoot, dry biomass of shoot and root *of* seedlings. 100 mg/L MWCNTs decreased the growth in *B. pendula*	*Betula pubescens & Betula pendula*	Zhuzhukin et al. 2023
AgNPs	10–30 μg/mL	Accelerated seedling growth	*Boswellia ovalifoliolata*	Savithramma et al. 2012
MWCNT–COOH	20 and 40 μg	Improved vigor of seedlings	*Alnus viridis* & *Shepherdia canadensis*	Ali et al. 2020
Silver & Copper nanoparticles (AgNPs & CuNPs)	5, 25 and 50 ppm	25 ppm increased root mycorrhization degree. 50 ppm disturbed the shape of plastids and starch content of seedlings	*Quercus robur*	Olchowik et al. 2017
AgNPs & CuNPs	5, 25 and 50 ppm	Stimulated root mycorrhizal formation and seedling growth. 25 and 50 pmm inhibited the formation of mycorrhizae in trees	Scots pine ( *Pinus sylvestris* )	Aleksandrowicz‐Trzcinska et al. 2018
AgNPs & CuNPs	5, 25 and 50 ppm	5 pmm increased maximum efficiency of photosystem II in *Pinus sylvestris* , while 25 and 50 ppm showed inhibitory impacts on growth of both species	*Pinus sylvestris* & *Quercus robur*	Aleksandrowicz‐Trzcinska et al. 2019
AgNPs	0, 10, 20, 40, 80 and 100 mg/soil kg & 0, 10, and 20 mg/L of water	No effect detected in low concentrations for both tree species. 80 mg/kg and 10 mg/L decreased length and dry matter in *Pinus sylvestris* seedlings	*Pinus sylvestris & Alnus subcordata*	Bayramzadeh et al. 2019
AgNPs	0.3 g/L	Increased viability, stimulated formation of roots, accelerated the growth of vegetative parts	Gray poplar ( *Populus × canescens* )	Vasyukova et al. 2021
FeNPs & CuNPs	2.10^−4^%–2.10^−2^%	Increased survival rate and annual height of seedlings	*Pinus sylvestris*	Polischuk et al. 2019
Zinc, iron, titanium, and copper oxide nanoparticles (ZnONPs, Fe_3_O_4_NPs, TiO_2_NPs and CuONPs)	400, 1200, 2000 mg/L for ZnO and Fe_3_O_4_ NPs 400, 600 and 1000 mg/L for TiO_2_ and CuO NPs	Increased survival rate, height, root diameter, stem fresh weight of seedlings	Scots pine ( *Pinus sylvestris* )	Ayan et al. 2023
Zinc and iron oxide nanoparticles (ZnO & Fe_3_O_4_ NPs)	30, 60 and 90 mg/L	Increased plant height, root length, leaf and branch numbers, shoot and root wights of tree species	*Moringa peregrina*	Soliman et al. 2015
TiO_2_NPs	50, 100, 200 and 500 mg/L	Increased fresh weight and root length of seedlings. concentration more than 100 mg/L induced some side effects	Camphor tree ( *Cinnamomum camphora* )	Zhou et al. 2022
AgNPs	200, 400, 600, 800, and 1000 mg/L	Caused decrease in plumula and radicle lengths	*Fagus orientalis*	Ozel et al. 2024
Fe_2_O_3_ NPs	2–10 mg/L	Gave more favorable results for the growth of seedlings	*Photinia fraserii* & *Cotinus coggygria*	Babali et al. 2024
ZnO NPs	0.5, 2.5, 5, 7.5 and 10 mg L^−1^	Increased photosynthetic pigments, bioactive compounds and enzymatic activity of seedlings	*Moringa oleifera*	Garza‐Alonso et al. 2021
AgNPs	0.2 mg	Enhanced growth of seedlings	*Moringa oleifera*	Azeez et al. 2019

In contrast, low concentrations of AgNPs showed no effects on the growth of *Pinus sylvestris* and *Alnus subcordata* (Bayramzadeh et al. 2019), while higher doses (80 and 100 mg/kg) negatively affected seedling length and dry biomass in *Pinus sylvestris* . Elevated concentrations of NPs have been found to suppress growth, inhibit mycorrhizal formation, and disturb the shape in several tree species (Olchowik et al. 2017; Aleksandrowicz‐Trzcinska et al. 2018; Zhou et al. 2022; Zhuzhukin et al. 2023). Furthermore, NPs applications have also led to adverse morphological effects, including reduced plumula and radicle length in *Fagus orientalis* seedlings (Ozel et al. 2024). Overall, the current understanding of how nanostructured materials influence tree growth remains limited and warrants further investigation.

### 2.2 Forest Protection (Stress and Risk Mitigation)

Recent research indicates that engineered nanomaterials and nanotechnology‐based biosensors have significant potential for the early detection of stress and risk factors in forest ecosystems. These include indicators related to soil moisture deficits, plant diseases, nutrient deficiencies, and wildfire threats (Yao et al. 2009; Chartuprayoon et al. 2010; Singh and Singh 2018; Wang et al. 2018; Wagay et al. 2023; Hontanon 2023). The application of nanostructured materials has been shown to mitigate the impacts of forest fires, water scarcity, salinity, environmental toxicity, and pest infestations throughout various life stages of tree species (Kashiwagi et al. 2005; Seeger et al. 2009; Sadati et al. 2020; Vasyukova et al. 2021; Mahdi et al. 2022).

#### 2.2.1 Drought Tolerance

The ongoing droughts driven by climate change are placing growing pressure on natural ecosystems and wild habitats, progressively altering ecological balances (Chiabai et al. 2018; Bowditch et al. 2020). Various CO_2_ emission scenarios predict that global warming will result in more intense and prolonged droughts by the end of the 21st century (Price et al. 2022; Chen et al. 2023). To enhance forestation and long‐term ecological restoration under environmental constraints, targeted innovative conservation interventions and a multi‐scale social‐ecological framework should be set to maintain the balance between social demands and ecosystem services (Yue et al. 2024).

Fortunately, modern nanotechnology offers promising tools to enhance vegetation's resilience to declining precipitation (Mohusaien et al. 2024). For instance, in experiments on hawthorn ( *Crataegus monogyna* ) tree seedlings, pre‐treatment with silica nanoparticles significantly improved physiological responses under drought stress, with notable enhancements in photosynthetic efficiency and stomatal conductance (Ashkavand et al. 2015). In arid regions where rainfall and water availability are limited, nanotechnology presents valuable opportunities for regreening and ecological restoration (Dorraji et al. 2010; Su et al. 2017; Giraldo et al. 2019). As demonstrated in Table 3, nanostructured and nanocomposite materials can enhance plants' tolerance to drought and other environmental stresses at physiological, morphological, biochemical, and molecular levels. Materials such as nanozeolite, multi‐walled carbon nanotubes (MWCNTs), and chitosan nanoparticles have been shown to improve tree resilience under water deficit and other harsh conditions (Sadati et al. 2020; do Carmo et al. 2021; Zhuzhukin et al. 2023). Additionally, nanoparticles of selenium, titanium, silicon, and silver have enhanced drought resistance in several tree species, including mango, pomegranate, and acacia (Mosa et al. 2022; Almutairi et al. 2023; Abdalkreem et al. 2025).

**TABLE 3:** Nanotechnology products applications in drought tolerance of forest and woody plant species.

Nanoparticles (NPs)	Concentration of NPs	Effects on tree	Tree species	References
Silicon dioxide nanoparticles (SiO_2_NPs)	10, 50, and 100 mg L^−1^	Increase resistance in photosynthesis and stomatal conductance of seedlings	Hawthorns (*Carataegus monogyna*)	Ashkavand et al. 2015
Multi‐walled carbon nanotubes (MWCNTs)	1, 10, 50, and 100 mg L^−1^	Increase expression of stress resistance genes. 100 mg/L concentration has negative effect on stress resistance genes	Birch ( *Betula pendula* ) tree	Zhuzhukin et al. 2023
(SiO_2_NPs)	10, 50 and 100 mg L^−1^	Pretreated with SNPs increased root length and biomass, improved nutritional status, photosynthesis, and stomatal responses to drought in seedlings	*Prunus mahaleb*	Ashkavand, Zarafshar, et al. 2018
Chitosan nanoparticles (NPs) containing S‐nitroso‐mer captosuccinic acid (S‐nitroso‐MSA)	0.2 mM	Improved drought tolerance habits of seedlings	*Heliocarpus popayanensis*	do Carmo et al. 2021
Selenium (Se), Titanium (Ti), and Silicon (Si) nanoparticles	5, 10, and 20 mg/L Se; 40, 60, and 80 mg/L Ti; and 50, 100, and 150 mg/L Si	Improved the growth attributes, yielding and fruit quality by reducing the effect of stressful conditions	Mango ( *Mangifera indica* )	Almutairi et al. 2023
SiO_2_NPs	1, 2, and 4 g	Increased total root length, root volume, root tips number and improved gas exchange under drought stress in seedlings	*Cunninghamia lanceolata*	Liu et al. 2023
Fe_2_O_3_‐graphitic carbon nitride nanostructures (Fe_2_O_3_/g‐C_3_N_4_)	0.2 gL^−1^	Improved physiological and biochemical resistance to drought	Olive tree ( *Olea europaea* )	Gholami et al. 2024
Silver nanoparticles (AgNPs)	5, 7.5 and 10 mL/L	Ameliorated shoot length, diameter, leaf chlorophyll, set of fruiting	Pomegranate tree ( *Punica granatum* )	Mosa et al. 2022
Nanozeolite	1% and 5% w/w	1% nanozeolite decreased the effects of water stress and improved growth of seedlings	*Populous euramericana triplo*	Sadati et al. 2020
SiO_2_ NPs	25, 50, 100, and 200 mg L^−1^	50 and 100 mg L^−^1 increased growth under drought stress in tree explants	*Malus domestica*	Avestana et al. 2017
SiO_2_ NPs	100, 300, 700, 1500, and 3000 mg L^−1^	Improved many physiological traits under drought stress	*Pistacia atlantica*	Mahmoodi et al. 2020
Ag NPs	25, 50 and 75 mg L^−1^	75 mg L^−1^ of AgNPs improved growth traits of seedling under drought	*Acacia senegal & Acacia mellifera*	Abdalkreem et al. 2025

#### 2.2.2 Salinity and Toxicity

The exposure of *Mangifera indica, Moringa peregrina*, *Eucalyptus tereticornis, Populus alba*, and *Salix alba* tree species to zinc, silicon, iron, magnesium, and titanium oxides nanoparticles (Table 4) increased the tolerance to salinity stress, reduced toxicity, and boosted tree quality (Seeger et al. 2009; Soliman et al. 2015; Fathy et al. 2019; Elsheery et al. 2020; Singh, Sillu, et al. 2021). It has been reported that hydrophilic polymers decreased the negative effect of soil salinity, minimized toxic components, and increased water holding capacity and nutrient use efficiency of plant, thus helping the success of plantation in arid and semi‐arid areas (Dorraji et al. 2010; Khan et al. 2017; Azeez et al. 2019; Al‐Mamun et al. 2021). Similar positive findings have been reported following the applications of selenium and silver nanoparticles in *Moringa oleifera* and *Phoenix dactylifera* tree subjected to different stressors of salinity and toxicity (Azeez et al. 2019; Mahdi et al. 2022). On the other hand, the use of high concentrations of nanoparticles such as zinc and silicon oxide nanoparticles under salinity conditions induces negative impacts on growth and nutrient uptake capacities of some woody plants (Elsheery et al. 2020). The use of various nanostructured materials with different concentrations in different tree species may elucidate necessary aspects of nanotechnology applications in saline and toxic conditions.

**TABLE 4:** Nanotechnology products applications in salinity and soil toxicity resistance of forest and woody plant species.

Nanoparticles (NPs)	Concentration of NPs	Effects on tree	Tree species	References
Nanoparticles of zinc oxide & silicon (ZnO NPs & SiNPs)	50, 100, and 150 mg/L of ZnONPs & 150 and 300 mg/L of SiNPs	Improved resistance, crop load and fruit quality under salinity. 300 mg/L of SiNPs showed negative effects on growth and nutrients uptake	*Mangifera indica*	Elsheery et al. 2020
ZnO & Fe_3_O_4_ NPs	30, 60 and 90 mg/L	Enhance salt tolerance capacity of tree	*Moringa peregrina*	Soliman et al. 2015
Fe_2_O_3_ NPs	25 ppm	Caused distinct biochemical changes hence salinity tolerance in tree	*Eucalyptus tereticornis*	Singh, Sillu, et al. 2021
Nanoparticles of Ferric Oxide & Magnesium Oxide (Fe_2_O_3_ NPs) & (MgO NPs)	3.0 and 6.0 mg/L each	Improved shooting and rooting abilities under salinity	*Populus alba*	Fathy et al. 2019
Titanium dioxide nanoparticles (TiO_2_ NPs)	1, 10, 20, 50 and 100 mg/L	No toxic impact shown on growth and physiological parameters of tree cuttings	Willow tree (*Salix* sp.)	Seeger et al. 2009
Silver nanoparticles (AgNPs)	5 mg	Degraded toxic environmental pollutants such as 4‐nitrophenol, 2‐nitrophenol and various hazardous dyes such as Congo Red, Methylene Blue and Methyl Orange up to 95%.	Not included	Shah et al. 2020
AgNPs	50 mL	Exhibited effective catalytic activity to degrade environment‐polluting dyes	Not included	Khatun et al. 2024
AgNPs	0.2 mg	Reduced toxicity of cadmium and lead in soil boosting growth of seedlings	*Moringa oleifera*	Azeez et al. 2019

#### 2.2.3 Pests and Diseases

Forests are at risk of huge losses due to disease outbreaks and invasion of pests for a wide range of plant species (Newsome and Noble 1986; Aleksandrowicz‐Trzcinska et al. 2018). However, recent studies have reported that these can be managed in a proper manner by nanomaterial applications (Table 5). Nanostructured materials have large‐scale potentials to be used as herbicides, insecticides, fungicides, and diagnostic tools to combat invasive pests and diseases of plants (Boonham et al. 2008; Chinnamuthu and Boopathi 2009; Yao et al. 2009; Baietto et al. 2010; Chartuprayoon et al. 2010; Bhagat et al. 2013). Many nanomaterials, including Coper–Carbon Core–Shell Nanoparticles (CCCSNs), are used as strong fungicides to medicate fungal infections in pine trees and empower wood resistance to termites and fungi (Qi et al. 2013). Silver, iron, copper nanoparticles improved the tolerance of many tree species against various infectious phytopathogens and pests (Polischuk et al. 2019; Vasyukova et al. 2021; Surendra et al. 2023; Abdalkreem et al. 2025). One important nanotechnology input in the forestry sector is the creation of disease‐resistant varieties of diverse plant species (Elhawat et al. 2018). Furthermore, according to Wagay et al. (2023) and Bhagat et al. (2013) the use of nanoparticles and nanocapsules in usually smaller doses as pesticides is more effective, ecologically beneficial, and has a high ability for plant protection.

**TABLE 5:** Nanotechnology products applications in pest and disease resistance of forest and woody plant species.

Nanoparticles (NPs)	Concentration of NPs	Effects on tree	Tree species	References
Iron and copper nanoparticles (FeNPs & CuNPs)	2.10^−4^%–2.10^−2^%	Increased peroxidase activity, inhibited catalase activity, thus increased resistance to infectious lodging in seedlings	*Pinus sylvestris*	Polischuk et al. 2019
Coper–Carbon Core–Shell Nanoparticles (CCCSNs)	0.1, 0.5, 1, 2.5 and 5 g/L	Improved wood resistance against decay fungi, blue stain fungi and Formosa termites	Pine (*Pinus* spp.)	Qi et al. 2013
Silver nanoparticles AgNPs	10, 25, and 50 μg/mL	Reduced Flacherie and Sappe microbial disease infecting *Bombyx mori* L. in tree leaves	Mulberry tree (*Morus* spp.)	Surendra et al. 2023
Copper and silver nanoparticles (CuNPs) & (AgNPs)	5, 15, 25, and 35 ppm	Showed antifungal activity in pathogens causing seedling damping‐off and wood decay in various tree species	Not included	Aleksandrowicz‐Trzcinska et al. 2018
AgNPs	0.3 g/L	Allowed obtaining 100% phytopathogen‐free explants in vitro tissue culture	Gray poplar ( *Populus × canescens* )	Vasyukova et al. 2021
AgNPs	25, 50 and 75 mg/L	Reduced seedling infection by *Rhizoctonia solani* fungi	*Acacia senegal & Acacia mellifera*	Abdalkreem et al. 2025

#### 2.2.4 Fire Control

Forest fires have become a critical environmental threat due to their detrimental impacts on global ecosystems and their potential contribution to climate change. In the western United States alone, approximately 150,247 wildfires occurred between 1992 and 2020 (Pourmohamad et al. 2025). Globally, the area affected by forest fires increased by about 5.4% between 2001 and 2023 (McCarthy et al. 2024). However, as shown in Table 6, advancements in nanotechnology present new opportunities to prevent the occurrence of wildfires or reduce the magnitudes of negative impacts. Understanding the applications and patterns of nanotechnology is essential for establishing effective strategies to mitigate and combat wildfires (Hontanon 2023; Carta et al. 2023). Nano‐based technologies such as Micro‐Electro‐Mechanical Sensors (MEMS) and various nanoparticles play a significant role in detecting, controlling, and suppressing wildfires (Olawoyin 2018; Hamdan et al. 2024). One notable example is MetFire Mesh, a proven nanotechnology solution that enables rapid flame extinguishing and promotes swift biological recovery in fire‐affected areas (Singh, Arya, et al. 2021).

**TABLE 6:** Nanotechnology products applications in wildfire control.

Nanoparticles (NPs)	Concentration of NPs	The effects	References
Aluminum Oxide nanoparticles (Al_2_O_3_)	0.45–2.45 g	> 0.6 g retarded ignition at fuel percentage of 11.4%. < 0.45 g didn't prevent ignition at fuel percentage of 11.4% > 0.8 g retarded ignition at fuel percentage of 12.1%. < 0.75 g didn't prevent ignition at fuel percentage of 12.1% > 1.3 g retarded ignition at fuel percentage of 13%. < 1.1 g didn't prevent ignition at fuel percentage of 13% > 2.45 g retarded ignition at fuel percentage of 14.8%. < 0.2 g didn't prevent ignition at fuel percentage of 14.8%	Hamdan et al. 2024
Ferrocene (FeCp_2_)	10^−4^ mol g^−1^	Suppressed fire flame in concentration between 0.1 × 10^−4^ to 9 × 10^−4^molg^−1^	Koshiba et al. 2012
Nano‐aluminum hydroxide (Nano‐ATH) particles	0.5%, 1%, 1.5%, 2%, 2.5%, and 3%	Increased ignition point, retarded flame, suppressed smoke, decreased sustained combustion time, decreased smoke production and heat release rates	Zhang et al. 2021
Silica hybrid foam (Silica nanoparticles)	30% pure SiO_2_, 6% SDS, and 64% H_2_O	Showed 50 times higher in fire extinguishing efficiency than that of ordinary water	Vinogradov et al. 2015
Multi‐walled carbon nanotubes (MWCNT)	0.05%–1.60% vol. in distilled water	Quenching time of liquid hydrocarbons by nanosuspensions was 3.5–5.0 times less than the time of quenching of liquid with finely divided water	Dali et al. 2020
The nanocomposite consists: inorganic magnesium hydroxide (MH), nitrogen‐based melamine cyanurate (MCA), and the phosphorus‐based ODOPB particles	MH 99.9% MCA 99.5% ODOPB 99%	More effective in fire extinguishing than other conventional powders in terms of extinction time and agent mass consumed	Ibrahim and Patruni 2020
Titanate nanotubes and graphene oxide composite (TNTs/GO)	2.5 wt.%	Improved the efficiency of flame retardancy and photodegradability of polyvinyl chloride	Sang et al. 2017
Single walled carbon nanotube (SWNT)	0.5 wt.%	Decreased flammability properties of polymer matrix	Kashiwagi et al. 2005
Fullerene‐decorated carbon nanotubes (C_60_‐*d*‐CNTs)	2.71 wt%	Conferred better flame retardancy to polypropylene	Song et al. 2009

Research by Hamdan et al. (2024) revealed that aluminum oxide nanoparticles used as fire inhibitors exhibited superior flame‐retardant properties compared to conventional materials, offering greater sustainability and less adverse environmental impacts. Likewise, the use of silica hybrid foam, nano‐aluminum hydroxide, and ferrocene nanoparticles has been shown to improve flame suppression, increase ignition point, and reduce both smoke production and heat release rates relative to traditional fire‐extinguishing agents (Koshiba et al. 2012; Vinogradov et al. 2015; Zhang et al. 2021). Additionally, various nanocomposites and nanostructured materials such as multi‐walled and single‐walled carbon nanotubes, as well as iron‐loaded polydopamine nanospheres have been found to enhance flame‐retardant efficiency (Sang et al. 2017; Dali et al. 2020; Ibrahim and Patruni 2020; Zhang et al. 2020). As climate change continues to elevate atmospheric temperatures and human activities exacerbate wildfire risks, research on advanced flame prevention and suppression techniques must be accelerated.

## 3 Trends and Implications of the Nanoparticles' Applications

**FIGURE 3:**
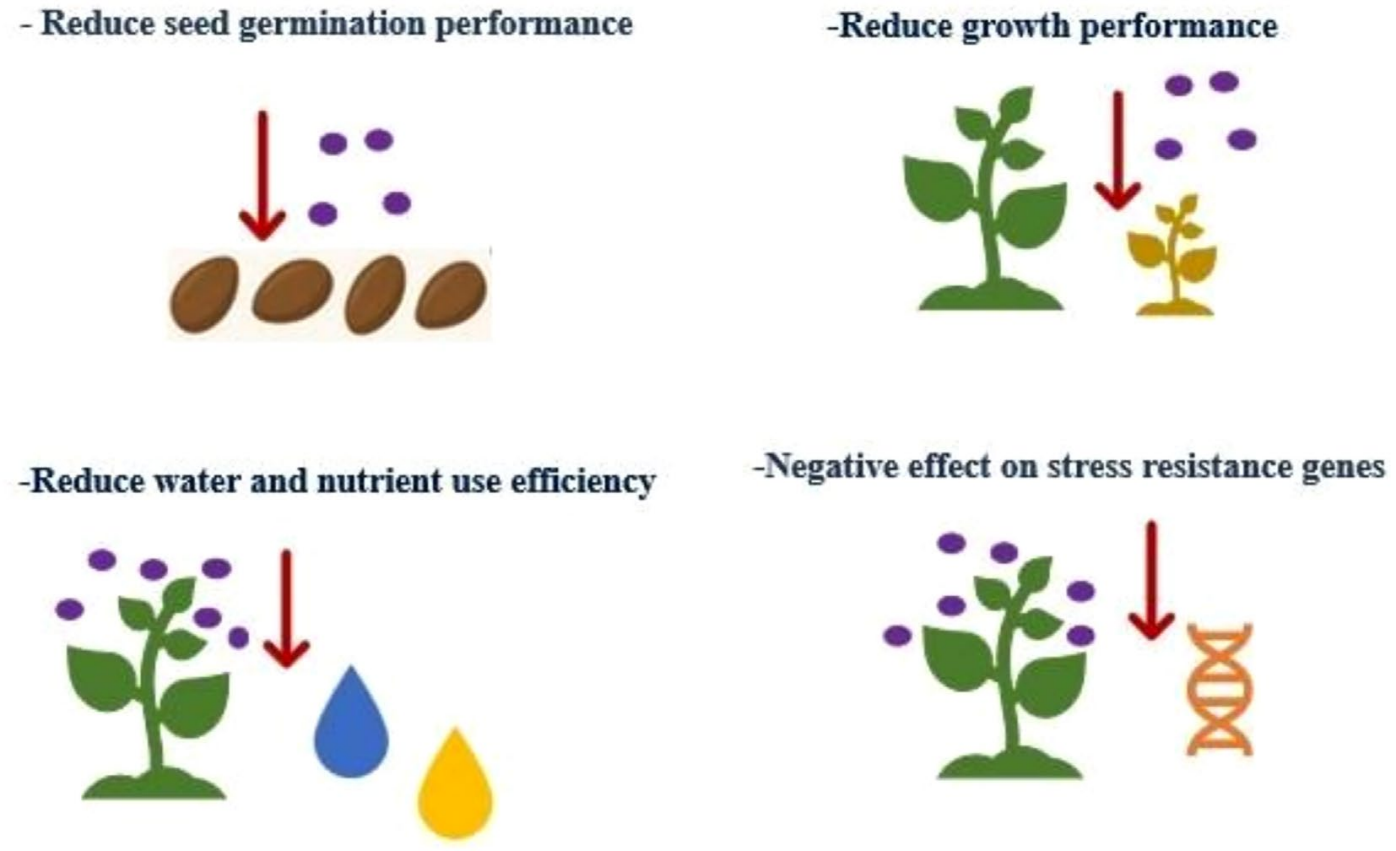
Negative impacts of nanoparticles' applications on seed and plant performance.

A comprehensive analysis of 50 academic published literature covering 64 research topics on the application of nanoparticles (NPs) in enhancing forest tree performance and resilience to stressful conditions reveals several notable trends and implications (Tables 1, 2, 3, 4, 5, 6). Based on current review, about 97% of NPs applications have induced positive effects on key parameters of germination, growth, and resistance to environmental stressors in trees and forest ecosystems. However, two studies reported divergent outcomes. Ozel et al. (2024) observed adverse impacts, while Bayramzadeh et al. (2019) found no effect of NPs on seed germination and seedling responses in *Fagus orientalis* and *Pinus sylvestris* respectively. Moreover, at high concentrations, NPs have been associated with negative effects in at least nine studies. As shown in Figure 3, it has been reported that the growth and physiological parameters in some plants are adversely affected by NPs' absorption. Approximately 50% of nanoparticle use in forestry has focused on enhancing seed germination (14%) and promoting the growth of seedlings and mature trees (36%). These aspects are foundational to successful silviculture, especially in the establishment and reforestation of stress‐prone environments. Improving seed germination and seedling performance is critical to the effective restoration of forest ecosystems (Raupp et al. 2020; Dias Laumann et al. 2023). The remaining 50% of NPs applications aim to enhance forest resilience to various abiotic and biotic stresses, including drought (18.6%), wildfire (13%), pests and diseases (8.6%), salinity (5.7%), and toxicity (4%).

In terms of tree species, only 17 families and 34 species have been involved in studies related to NPs applications. These represent only about 7% of global tree families and a mere 0.05% of known tree species. Globally, there are approximately 261 botanical tree families (Kindt et al. 2023); encompassing around 73,300 species (Cazzolla Gatti et al. 2022). This limited scope aligns with observations that, despite the promising potential of nanotechnology in forestry, its application remains underexplored in forest ecosystems and woody plants (Wagay et al. 2023). Thus, there is a pressing need to review existing literature, identify research gaps, and conduct further experimental studies to support the sustainable integration of nanotechnology in forestry practices (Siam et al. 2025). The most commonly studied tree families include *Betulaceae, Rosaceae*, and *Salicaceae*; each represented by four species. These are followed by *Moringaceae, Pinaceae*, and *Anacardiaceae*; each with three species examined. The focus on these families appears to be influenced by their domestic socioeconomic importance. For instance, species from *Betulaceae* (*Betula* and *Alnus* spp.), *Salicaceae* (*Populus* and *Salix* spp.), and *Pinaceae* (*Pinus* and *Larix* spp.) are widely utilized for timber and traditional medicine. Similarly, *Rosaceae* species (*Crataegus*, *Photinia*, *Prunus*, and *Malus* spp.), *Moringaceae* ( *Moringa oleifera* and *Moringa peregrina*), and *Anacardiaceae* ( *Cotinus coggygria* and *Mangifera indica* ) are commonly used as food sources and for medicinal applications. The socioeconomic significance of these species positions them at the center of preservation and sustainable use strategies (Bett et al. 2021). Forest tree conservation plays a vital role in providing biogenic resources for NPs fabrication, which also could be used to promote environmental and societal services, including forestry (Khatun et al. 2024). It is therefore essential that future nanotech‐based improvement investigations in forestry expand to include a broader range of tree families and species on the earth. Notably, research on wildfire resilience has mostly focused on how ecosystems respond to fires, rather than how individual tree species are affected. Studies in this area show that nanoparticles can change fire‐related properties, such as making it harder for fires to start, slowing down combustion, reducing smoke, and decreasing heat release. There is still a need for more targeted research on using nanotechnology to mitigate wildfires, both at the ecosystem level and for individual tree species. Overall, expanding research on how nanotechnology can work together to promote tree growth, restore ecosystems, and increase resilience to environmental stress should be a priority to ensure long‐term ecological sustainability and societal well‐being.

Regarding the types of nanoparticles used, Tables 1, 2, 3, 4, 5, 6 show the use of a variety of types and concentrations of NPs across various forest tree species. These NPs can be classified into metallic, metal oxide, carbon‐based, and hybrid or composite nanoparticles, showcasing considerable differences in their frequency and functional roles.

- Silver Nanoparticles (AgNPs): silver nanoparticles were the most frequently reported, appearing in 22 studies, which is approximately 42% of all reviewed entries. AgNPs have been utilized across multiple domains, including seed germination, seedling growth, drought and salinity tolerance, toxicity mitigation, and pest and disease resistance studies. Their popularity stems from their strong catalytic, antimicrobial, and antioxidant properties, making them effective in enhancing plant vigor and resistance. However, their usage is not without concern; several studies (e.g., Bayramzadeh et al. 2019; Ozel et al. 2024) reported negative impacts at higher concentrations, such as reduced germination and inhibited growth, underscoring the importance of concentration control.
- Zinc, Silicon, Iron, and Copper‐Based Nanoparticles: following AgNPs, Iron‐based nanoparticles, including Fe_2_O_3_, Fe_3_O_4_, and general iron formulations (FeNPs), appeared in nine studies (17% of studies). These NPs contributed to seedling growth, drought, and salt tolerance. Zinc oxide nanoparticles (ZnO NPs) were identified in four studies (Soliman et al. 2015; Elsheery et al. 2020; Garza‐Alonso et al. 2021; Ayan et al. 2023), used primarily to improve seed germination, stimulate photosynthetic activity, and mitigate salinity effects. Their ability to enhance enzymatic activity and nutrient uptake supports their frequent inclusion in studies related to growth and stress. Silicon and silicon dioxide nanoparticles (SiO_2_ NPs) were present in six studies (Ashkavand et al. 2015; Avestana et al. 2017; Ashkavand, Zarafshar, et al. 2018; Mahmoodi et al. 2020; Liu et al. 2023; Almutairi et al. 2023), mostly related to drought and salinity tolerance. SiO_2_ NPs are known for enhancing root growth, gas exchange, and water retention in plants under abiotic stress conditions. Copper nanoparticles (CuNPs) were reported in five studies (Qi et al. 2013; Aleksandrowicz‐Trzcinska et al. 2018; Polischuk et al. 2019; Ayan et al. 2023) (approximately 9.4%) for enhancing plant health, increasing root mycorrhization, and demonstrating antifungal and photosynthetic benefits.
- Titanium, Magnesium, and Selenium Dioxide and Metals Nanoparticles: titanium dioxide nanoparticles (TiO_2_ NPs) were observed in four studies (Seeger et al. 2009; Zhou et al. 2022; Almutairi et al. 2023; Ayan et al. 2023), primarily used for enhancing seed germination, stimulating growth, and alleviating salinity stress. These NPs are valued for their photocatalytic activity and their role in regulating oxidative stress. Additionally, magnesium oxide (MgO) and selenium (Se) nanoparticles appeared sporadically in one to two studies each, indicating their emerging but underexplored potential in forestry applications.
- Underrepresentation of Carbon‐Based Nanomaterials: despite their extensive use in other fields, carbon‐based nanomaterials such as multi‐walled carbon nanotubes (MWCNTs), single‐walled carbon nanotubes (SWCNTs), graphene oxide (GO), and fullerenes have only been documented in 7 studies (~13%). These materials were primarily applied to enhance seedling growth, drought tolerance, and wildfire suppression due to their structural strength and electrical conductivity. However, their limited use in forest‐specific applications indicates a significant gap in forestry nanotechnology research, especially considering their functional versatility.
- Hybrid, Composite, and Fire‐Resistant Nanoparticles: hybrid nanocomposites and specialized formulations, such as nano‐aluminum hydroxide (Nano‐ATH), ferrocene, magnesium hydroxide‐based composites, and silica foams, were mainly highlighted in wildfire mitigation studies. These materials improved ignition resistance, minimized smoke and combustion duration, and enhanced flame retardancy. Despite their promising outcomes at the ecosystem level, studies seldom addressed tree species‐specific effects, highlighting the need for more focused research in this area.

In summary, the reviewed literature demonstrates a strong preference for metallic nanoparticles, particularly those based on silver, zinc, silicon, and iron, which collectively account for the majority of forestry applications. While over 85% of reported applications yielded positive outcomes, at least 9 studies (~17%) recorded neutral or negative effects, typically at high concentrations or in sensitive species. The underutilization of carbon‐based and green‐synthesized NPs, as well as the limited focus on long‐term ecological impacts and species‐specific responses, highlight key research gaps. For nanotechnology in forestry to be sustainable, future studies should look at a wider range of nanoparticle types, find the best application concentrations, and standardize safety and environmental assessments. It is also crucial to expand research to less‐studied tree species and stress conditions to get the most benefits from nanotechnology in forest ecosystems.

## 4 Synergistic Approach Needs for Integrating Nanotechnology and Forestry

Given the significant importance of forests as a renewable biodiverse resource and nanotechnology as an innovative and rapidly advancing field, there is a need for a synergistic management approach including research, policy, and advocacy. This approach of integration of nanotech in forestry practices should aim to generate sufficient data to inform sustainable management practices. As forests serve both as a source for the green synthesis of nanoparticles and as a platform for their application, it is essential to establish standardized protocols for biosynthesis and use across different tree species and forestry activities (Singh, Sillu, et al. 2021; Vasyukova et al. 2021; Irewale et al. 2024; Khatun et al. 2024). Developing such protocols requires a global understanding of NPs composition, synthetic biology, bioengineering techniques, application methods, dosage, and timing (Korbekandi et al. 2015; Myburg et al. 2019; Bett et al. 2021). Collaborative research and field trials integrated with monitoring, feedback mechanisms, reporting, and the engagement of local communities, educational institutions, and policymakers are essential at both national and international levels to create effective agricultural and forestry management strategies (Jerson and Hinckley 2023; Raman et al. 2024). Well‐structured and comprehensive research is key to identifying knowledge gaps and assessing the impacts of nanotechnology in the forestry sector. This includes understanding biosynthesis mechanisms, nanoparticle types, beneficial and adverse effects, and optimal application timing to enhance tree growth, physiology, morphology, stress resilience, soil properties, and wood product quality across various tree species and families.

Future research should address several fundamental questions, including: To what extent can nano‐based fertilizers, growth promoters, and pesticides enhance growth performance, improve resilience to environmental stresses, and protect trees from pests and diseases? How effectively can nanosensors monitor forest health and detect early signs of drought, salinity, or wildfires, thereby enabling precision forestry and promoting sustainability while reducing ecological degradation? Can forest‐derived nanomaterials replace petroleum‐based materials in sectors such as packaging, construction, and textiles, thus promoting renewable biomaterials and reducing carbon footprints? Can nanotechnology facilitate the conversion of forest residues and wood waste into high‐value bio‐based products, supporting a circular economy in the forestry sector and minimizing environmental harm? To what degree can nanomaterials enhance the strength, durability, water resistance, fire retardance, and carbon storage capacity of wood products, thereby extending their lifecycle and reducing raw wood consumption? Will investing in emerging nanotechnologies ensure the forestry sector remains globally competitive, particularly in high‐value, environmentally friendly markets, while generating green technology‐based employment? Can the potential negative consequences of nanotechnology be precisely identified and mitigated to prevent harm to the environment, humans, animals, and plants? What are effective strategies for integrating the cultural and socioeconomic characteristics of local communities into forestry‐based nanotechnology initiatives? Answering these questions requires the scientific community to broaden its research scope and efforts. Bridging current knowledge gaps will facilitate the development of effective policies and conservation actions that support sustainable forestry practices (Raman et al. 2024). Similarly, advocacy efforts are needed at global, national, and individual levels to promote sustainable management and stress resilience of forests (Ma et al. 2022; Liu and Yu 2023).

On the other side, in consideration of the current trends and outcomes outlined in this review, the prospective application of nanotechnology in the field of forestry should emphasize the optimization of widely utilized nanoparticles, including silver (AgNPs), zinc oxide (ZnO NPs), silicon dioxide (SiO_2_ NPs), and iron‐based NPs. These nanoparticles have consistently exhibited positive impacts on seed germination, seedling growth, and stress tolerance. Nevertheless, the recurrent adverse effects observed at elevated concentrations, particularly concerning AgNPs, underscore the necessity for precise dosage protocols. Additionally, underexplored nanomaterials such as carbon nanotubes and nanozeolites, which have demonstrated potential in enhancing drought resilience and facilitating fire suppression, necessitate further investigation. Research in the field of carbon nanomaterials is ongoing to characterize their potential applications across various domains (Aggrawal et al. 2024). Future initiatives should involve species‐specific applications, standardized concentration ranges, the integration of nanosensors for stress detection, and a comprehensive investigation into environmentally friendly, forest‐derived NPs to promote sustainable and resilient forestry practices.

## 5 Conclusions

The analysis of academic literature revealed that the application of nanoparticles as pre‐treatment stimulants has significantly enhanced seed germination, seedling growth, and survival rates by breaking seed dormancy and improving water‐holding capacity and nutrient use efficiency. Nanoparticles and nanocomposites also contribute to increased resilience in trees and forests against drought, salinity, toxicity, pests, diseases, and forest fires. The review identified 17 tree families and 34 species studied in relation to nanoparticle applications. The selection of these families appears to be influenced by their domestic socioeconomic importance. Approximately 50% of nanoparticle use in forestry has been directed toward enhancing tree growth and seed germination, while the remaining 50% has focused on improving forest resilience to environmental stressors. However, adverse effects, particularly at high nanoparticle concentrations, on seed germination and tree growth have also been reported. Given the critical role of forests as renewable biological resources and the rapid advancement of nanotechnology, a coordinated global research effort is essential. The future of nanotechnology research holds incredible promise, particularly when it comes to enhancing forest health monitoring. By integrating advanced nanosensors and innovative nanomaterials, we can revolutionize our ability to detect early signs of stress, anticipate disease outbreaks, and identify environmental contamination. This cutting‐edge approach will not only help us assess wood quality but also uphold the integrity of entire ecosystems, ensuring that our forests thrive for generations to come. For sustainable nanotechnology research in forestry, it is imperative to expand applications across a wider range of tree species and families and to adopt interdisciplinary, collaborative approaches involving diverse scientific and development sectors.

## Funding

The authors have nothing to report.

## Conflicts of Interest

The authors declare no conflicts of interest.

## Data Availability

The authors have nothing to report.
